# Material properties of the skull layers of the primate parietal bone: A single-subject study

**DOI:** 10.1371/journal.pone.0229244

**Published:** 2020-03-03

**Authors:** Uriel Zapata, Qian Wang

**Affiliations:** 1 Department of Mechanical Engineering, EAFIT University, Medellín, Colombia; 2 Department of Biomedical Sciences, Texas A&M University College of Dentistry, Dallas, TX, United States of America; Ohio State University, UNITED STATES

## Abstract

The outer cortical table of the parietal bone has been commonly used as a calvarial bone graft site for the craniofacial reconstruction. However, little is known about how removing the outer table may affect the function and structure of the inner table, and how the knowledge of the biomechanics and material properties of cortical bones will help the calvarial graft to better integrate into the biological and mechanical functions of its surrounding native tissues. In this study, it was hypothesized that there were significant differences in both density and material properties between inner and outer cortical plates in cranial bones. Twelve cylindrical specimens, including inner-outer layers, of cortical parietal bone of a female baboon were collected. Cortical thicknesses and densities were measured, and elastic properties were assessed using an ultrasonic technique. Results demonstrated remarkable difference in both thickness (t = 8.248, p ≤0.05) and density (t = 4.926, p≤0.05) between inner and outer cortical paired samples. Orthotropic characteristics of the cortical plates were detected as well, these findings suggest that there are differences in biomechanical properties between two surfaces of cranial bones at both tissue and organ levels. How these differences are linked to the stress environments of the inner and outer cranial cortical layers awaits further studies. Further study will greatly enhance our ability to address questions derived from both morphological and craniofacial medicine fields about the development and biomechanics of craniofacial skeletons.

## Introduction

The skull is a complex arrangement of bones, joined by sutures, forming the craniofacial region; this craniofacial complex is comprised of 22 facial and cranial bones of different shapes, thicknesses, and functions. The cranial shape is the result of several functional factors such as: (1) the neurocranial growth which produces osseous expansion, (2) the location, morphology, and patency of cranial sutures, (3) the presence of supraorbital ridges to enforce the connection between the orbits and the brain case, and (4) the three-layered structure of the cranial bone which is composed of an outer cortical table (periosteal cortical plate), a low density core known as the diploe and an inner cortical table (endosteal cortical plate) [1]. The cranial bones are a three-layered system with two external cortical plates packing a thin layer of trabecular bone. The origin of the three cranial layers is an intramembranous ossification process. First, mesenchymal cells produce the ossification centers, and then osteoid is secreted within the fibrous membrane to form two layers of compact bones, ectocranial table (outer plate) and endocranial table (inner plate), and a central cavity containing red marrow (diploe) [2].

Overall, the cranial vault protects the brain from physical injuries, with a dome-shaped three-layered structure. It has been suggested that neurocranial growth produces the outer layer of the dura becoming the inner endosteal layer of calvarial bones [1]. That is, the shape, the thickness and the mechanical properties of the inner surface of the osseous cranial vault are a direct reflection of either, the growth rate of the brain at early ages or the cerebral shrinkage at old ages. On the other hand, the outer periosteal table of the calvarial bones not only is in contact with the external environment, but also provides the support for both masticatory and nuchal muscular attachments [3]. Based on this disparate function of the two calvarial layers, the inner and the outer cranial plates are expected to exhibit different mechanical properties, possibly associated with loads resulting from diverse orofacial functions [4].

The outer cortical table of the parietal bone has been commonly used as a calvarial bone graft site in craniofacial reconstructions [5, 6]. However, little is known about how the removal of the outer table might affect the function and structure of the inner table, and further, how the knowledge of the microstructure and material properties of cranial bones could improve the design of a graft that can integrate better to the biological and mechanical function of its surrounding native tissues [7]. In this regard, the biomechanical relationship between the outer and inner cortical tables in the parietal bone is not clear: Are they coupled or not related at all in terms of bone function and adaptation? An in-depth comparative study into the microstructure of the cortical bones at the same location in the parietal bones, occupying the ectocranial and endocranial surfaces, is likely to answer these questions.

Recent work has demonstrated that the three-dimensional material properties of cortical bone vary throughout the craniofacial skeleton [8–10]. For instance, there is a general correlation of bone anatomical axis and orientations of bone maximum stiffness at some areas, such as the supraorbital torus and zygomatic arch, suggesting a link between bone mechanical properties and structures at the tissue level [11, 12]. Furthermore, the coupling of micro-CT imaging and ultrasonic work on the cortical bone layers of the human mandibles and femurs [13] proved the important relationship between the bone anisotropy of material properties and the spatial configuration of the osteonal system, which is also supported by our recent study on baboon mandibles [14]. Our finding represent a new understanding of the structural basis of bone mechanical properties at the tissue level, in which the long axes of the osteons, as represented by the Haversian canals, are aligned along to the axes of maximum elastic stiffness (or E_3_) of cortical bone (Explanations of elastic properties are detailed below in materials and methods section).

Several studies have examined the overall mechanical properties of the cranial vault as a whole by testing the mechanical properties of the vault using: tension test [5, 16], compression test [5, 7, 9], triaxial compression test [7], shear test [7], torsion test [7], three point bending test [16–22], four point bending test [23],and simple-bending test [24]. However, the results of these studies represent only the overall mechanical properties of all three layers.

Many have studied the cortical material properties of the craniofacial complex in mammals, including humans [10, 14–20], yet only a few studies have addressed the differences of mechanical properties between external and internal cortical plates. Evans and Lissner (1957) performed both tension and compression tests of the cortical layers of human parietal bone separately [21]. Unfortunately, the compression test was performed on specimens which had been used for a previous tension test, affecting their initial mechanical conditions. Later, Dempster (1967) examined the cortical grain patterns for the inner and outer table of the human brain case using split-line methods in six complete human skulls [22], and he reported different random pattern distributions of fibrous matrix in both the inner and the outer plates of the skull vault, suggesting the layers have different mechanical properties, but also tangential isotropy to the skull surface. McElhaney and collaborators (1970) performed Vickers microhardness and tension tests on both inner and outer layers of bones from both humans and *Macaca mulatta* and reported no significant differences in hardness or tensile properties between the two plates [23]. Wood (1969, 1971) tested the two cortical plates of parietal, frontal and temporal human cranial specimens using tension test of bone specimens parallel to both the sagittal and the coronal sutures [15, 24], and he reported no significant differences in elastic modulus between the two cranial cortical plates. Hubbard (1971) tested the three components of the human calvarial system by using both four and three point bending tests, and he reported no significant differences in flexural response in any of several orientations [25]. Peterson and Dechow (2002) reported the mechanical properties of the inner and outer tables of the human parietal bone tested with an ultrasonic technique and reported significant differences in both mechanical properties and densities between inner and outer cortical tables [3].

The layered composition of the three-layered calvarial vault suggests independent biomechanical purposes for each cranial stratum. However, there is little information regarding how the material properties of each layer are correlated to maximize its capabilities to undertake functional loadings. In this study, the density and elastic properties of two cortical plates of the parietal bone were studied in a baboon skull. We hypothesized that bone density and elastic properties are different between two cortical plates of the parietal bone due to orofacial functional differences.

## Materials and methods

**Fig 1:**
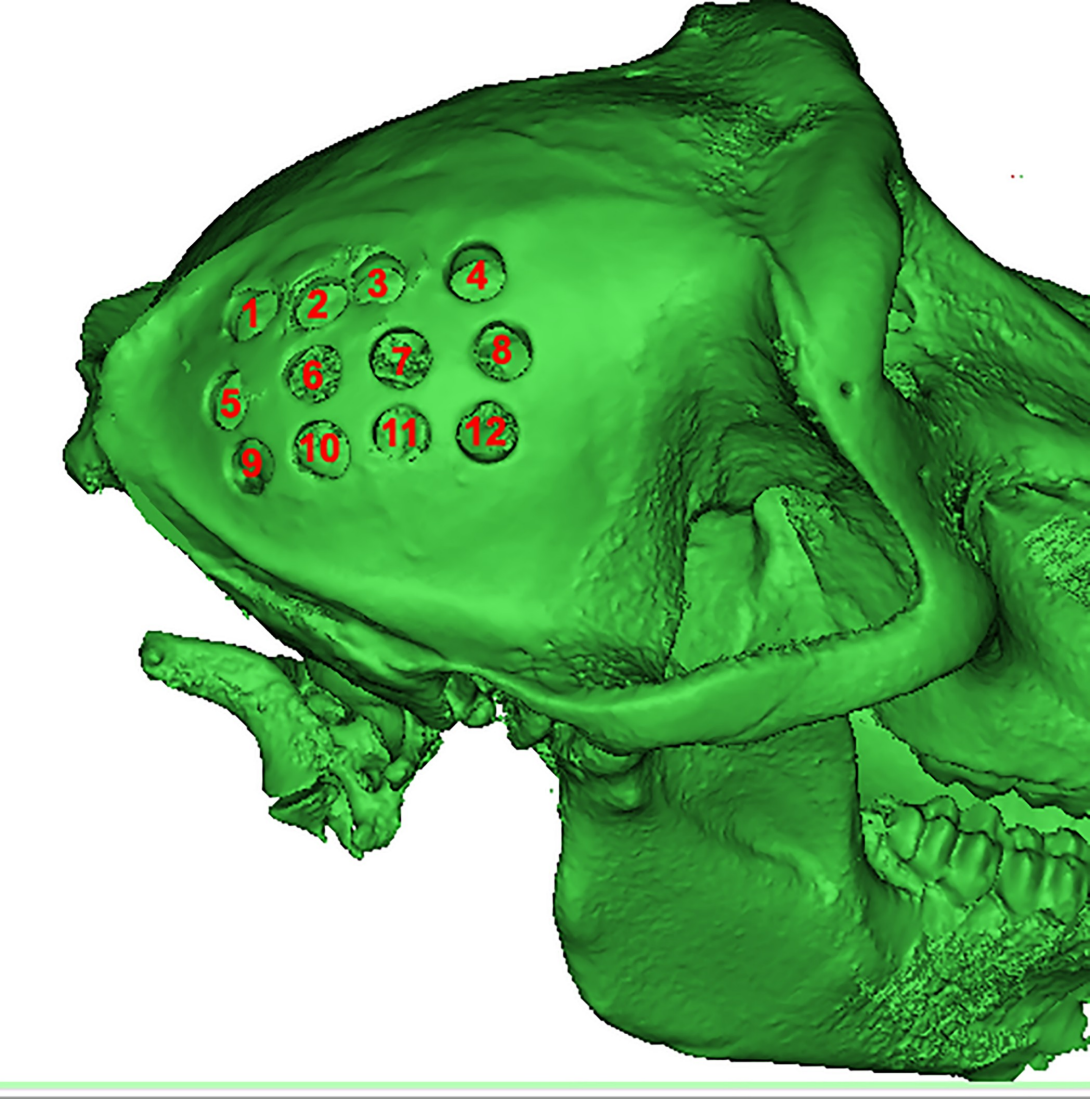
Location of the sampling sites on the right parietal bone.

The animal tissues were obtained from 14-year-old female baboon (sample reference # 10881) from the Southwest National Primate Research Center (supported by NIH-NCRR P51 RR013986). Animal tissues were handled according to the NIH, Federal, State and local rules and regulations. The fresh-frozen tissue was stored in a freezer at -20°C before the removal of bone samples. It has been reported that the freezing process of hard tissues has a minimal effect on the elastic properties of cortical bone [26, 27]. After removing skin and muscle, twelve cylindrical cortical bone specimens (Fig 1), 5 mm in diameter, were extracted from the right parietal bone using a low-speed rotary tool (Dremel, Model 732, heavy duty flex shaft, Bosh Inc., Germany) under permanent irrigation with saline solution. Prior to the removal, the cylindrical cortical specimens were marked with a central graphite line parallel to the sagittal suture, to ensure a common reference system for all the specimens. After the removal of each cylindrical sample the core was split, leaving two cortical plates with attached trabecular structure in each of the sides. The trabecular remnants were removed from each cortical sample by grinding the surfaces with a water-cooled grinding machine (Tormek, SuperGrind 2000, Linidesberg, Sweden). The cortical samples from both outer and inner plates were stored in a 50:50 solution of 95% ethanol and isotonic saline solution at room temperature (21°C) in order to maintain their mechanical properties [28].

**Fig 2:**
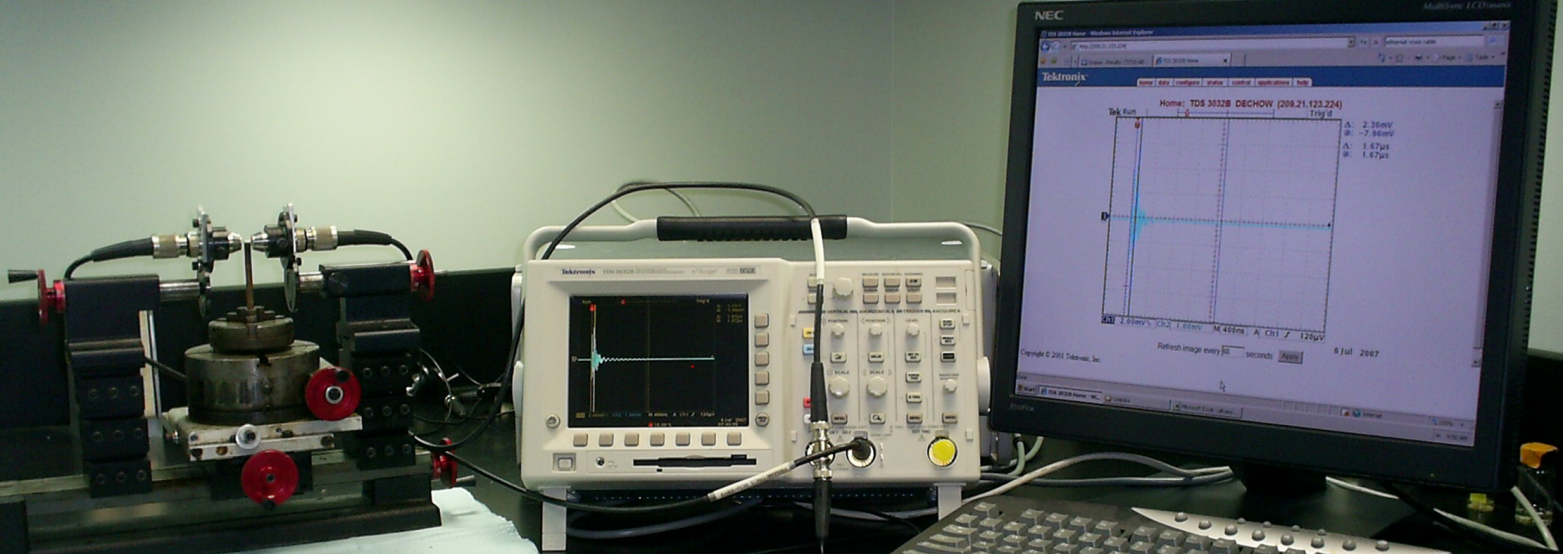
Ultrasonic system used to test mechanical properties in cylindrical cortical bone specimens.

Both the cortical thickness and the diameter of each cylindrical specimen were measured three times, in different directions, using a digital caliper to ensure reliability and decrease error. In the same way, the apparent density, based on Archimedes´ principle of buoyancy, was measured three times for each cortical specimen, corrected by the effect of temperature, and the mean values were recorded [28]. The elastic mechanical properties of each specimen were assessed using an ultrasonic technique [28, 29]. Longitudinal ultrasonic waves were produced by a pair of V312-SU, 10 MHz, longitudinal piezoelectric transducers (Olympus Inc, Waltham, MA), while transverse ultrasonic waves were generated by a pair of V156-RM, 5 MHz, shear piezoelectric transducers (Olympus Inc, Waltham, MA). The waves were sent through each cylindrical cortical bone sample in nine radial directions, with 22.5° intervals around the external circumference of the specimen, as shown in Fig 2. In addition, one set of waves was delivered perpendicular to the cortical specimen. The time delay of the perpendicular path was combined with the caliper thickness of the specimens, whereas the time delay of the radial path was complemented with the diameter of the specimens to calculate the ultrasonic velocity of the waves through the cortical material. Elastic material properties were obtained from the principles of linear elastic wave theory based on Hooke´s law using velocities, densities, and thicknesses [28]. All the measurements were performed for the same operator.

Several elastic material properties were obtained: (1) principal elastic moduli E_1_, E_2_, and E_3_, which represent the ability of a system to resist axial deformation by a given load, (2) principal shear moduli G_12_, G_23_, and G_13_, which embody the ability of a system to resist shear deformation produced for an external load, (3) Poisson´s ratio ν_12_, ν_23_, and ν_13_ which represent the ability of a structure to resist normal strains in perpendicular directions, (4) orientation of the axis of maximum stiffness (in degrees), relative to the graphite line drawn parallel to the sagittal suture before extraction of the cortical specimen, and (5) anisotropy quantified as the ratio between one of the minimum elastic modulus and the maximum elastic modulus (E_2_/E_3_). Values of anisotropy close to 1.0 suggest an isotropic condition of the material, whereas values less than 1.0 indicate the material tends to be orthotropic.

### Statistics

Statistical evaluation of the data were performed using IBM SPSS statistical software (SPSS, Version 25, IBM, Armonk, New York). Descriptive statistics were performed on all the independent variables. A Kolmogorov-Smirnov test of normality was applied to the independent variables to assess the normality of the distributions. In addition, a Levenes´s test was performed to check if the variances of the outputs for the inner and the outer plates were approximately equal. A paired t-test was used to search for differences between inner and outer mechanical properties. The hypothesis requires a two-tail test, with alpha value α = 0.05, and 11 degrees of freedom; thus, the critical value for the level of significance was stated as CV = ±2.201. The angular orientation of the axis of maximum stiffness were evaluated with circular descriptive statistics using Oriana statistical analysis software Version 2.0 (Kovach Computing Services, Pentraeth, UK). Differences between paired multiple angular means were calculated using the Watson-Williams F-test.

## Results

Descriptive results are reported as the mean and the standard deviation (Table 1). Most of the variables had normal distribution, except the density of the outer layer D(12) = 0.259, p = 0.026, and the density of the inner layer D(12) = 0.266, p = 0.019. Thus, Wilcoxon test was used to search for differences in density between inner and outer cortical plates. Levene´s test showed equal variances for all the variables.

**Table 1:** Elastic mechanical properties for outer and inner cortical plates.

Property	Mean	Std. Dev.
Thickness Outer (mm)	1.98	0.35
Thickness Inner (mm)	1.42	0.25
Density Outer (kg/m^3^)	1939.84	26.23
Density Inner (kg/m^3^)	1911.60	26.72
E_1_ Outer (GPa)	16.29	1.07
E_1_ Inner (GPa)	14.23	1.33
E_2_ Outer (GPa)	21.84	1.17
E_2_ Inner (GPa)	21.22	0.82
E_3_ Outer (GPa)	28.05	1.14
E_3_ Inner (GPa)	24.14	2.03
G_12_ Outer (GPa)	7.72	0.46
G_12_ Inner (GPa)	6.84	0.51
G_31_ Outer (GPa)	8.40	0.55
G_31_ Inner (GPa)	7.04	0.73
G_23_ Outer (GPa)	10.89	0.97
G_23_ Inner (GPa)	9.99	0.97
ν_12_ Outer	0.21	0.04
ν_12_ Inner	0.24	0.06
ν_31_ Outer	0.20	0.05
ν_31_ Inner	0.25	0.07
ν _23_ Outer	0.13	0.07
ν _23_ Inner	0.14	0.08

**Fig 3:**
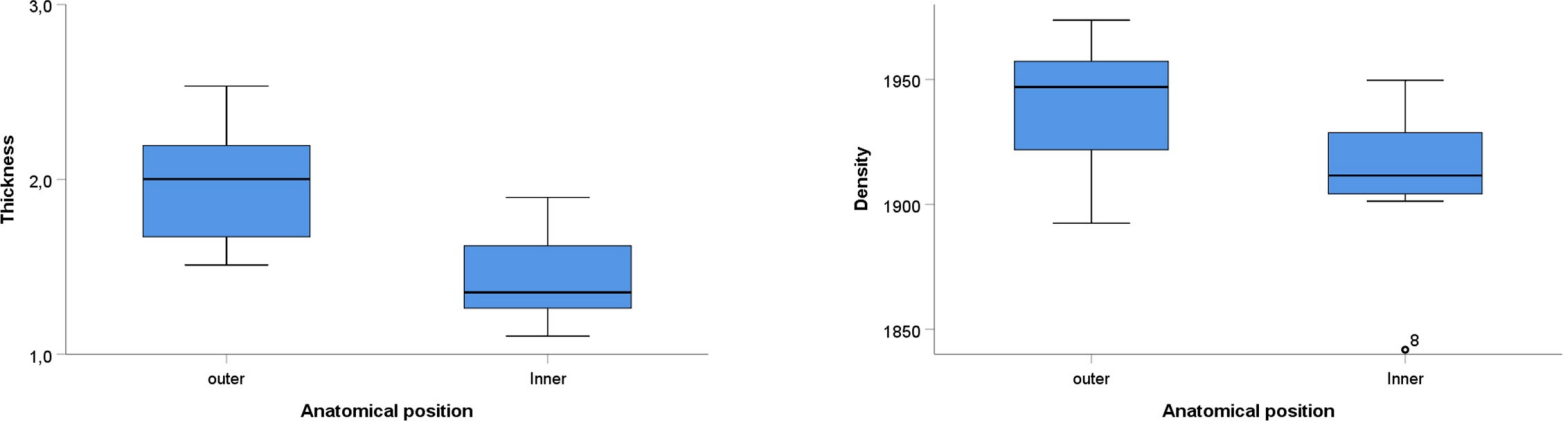
Differences in thickness (mm), and densities (kg/m^3^) between inner and outer cortical cranial plates.

Overall, results show statistical differences in both thickness (t = 8.248, p ≤ 0.05) and density (Z = -2.824, p = 0.005) between paired samples of inner and outer cortical plates of the parietal bone (Fig 3). The outer table was both thicker and denser than the inner table. Thicknesses of the inner and outer plates were not correlated (R = 0.743, p = 0.06), nor were the densities of the tables (R = 0.719, p = 0.08).

**Fig 4:**
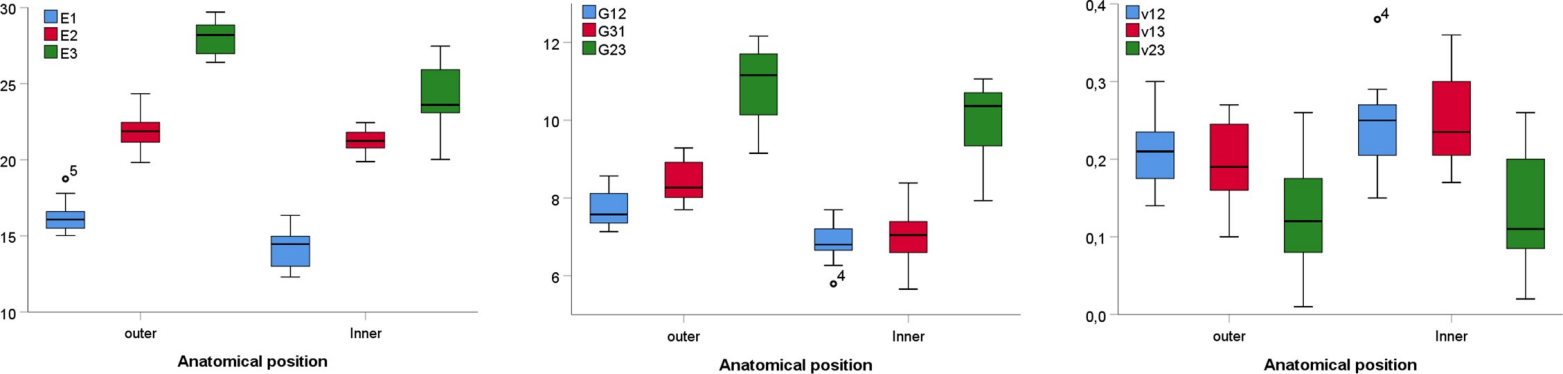
Principal elastic mechanical properties for baboons in the three-layered calvarial system. (A) Elastic moduli, (B) shear moduli, and (C) Poison´s ratios.

Significant differences for minimum elastic modulus E_1_ (t = 5.332, p < 0.05) and maximum elastic modulus E_3_ (t = 6.321, p < 0.05) were found between inner and outer cortical plates (Fig 4A). However, there were no statistical differences in principal elastic modulus E_2_ between the two cortical plates. Specifically within the outer cortical table, statistical differences were found between E_1_ and E_2_ (t = -11.543, p < 0.05) and between E_2_ and E_3_ (t = -16.683, p < 0.05). Similarly, significant differences were found between E_1_ and E_2_ (t = -13.784, p < 0.05) and between E_2_ and E_3_ (t = -5.006, p < 0.05) for the inner cortical plate. For the shear moduli, significant differences were found for G_12_ (t = 5.455, p < 0.05), G_31_ (t = 6.466, p < 0.05), and G_23_ (t = 2.876, p = 0.015) between inner and outer cortical plates (Fig 4B). The Poison´s ratio ν_31_ was significantly different between inner and outer cortical plates (t = -2.418, p = 0.034). There were no statistical differences in either ν_12_ and ν_23_ between cranial cortical plates (Fig 4C).

**Fig 5:**
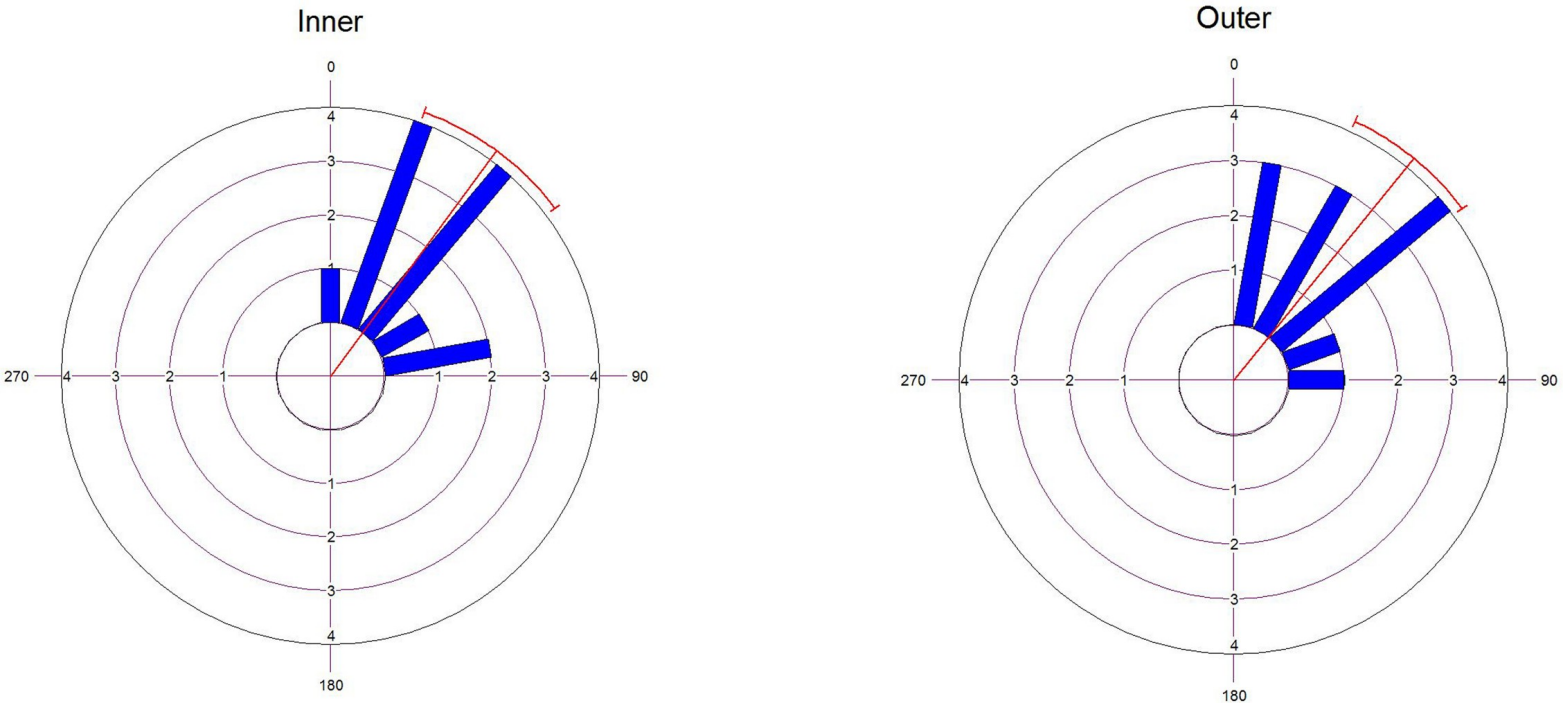
Angular orientation of the axis of maximum stiffness for both cortical plates in the baboon´s skull. The direction 0 matches the direction of the sagittal suture.

Angular orientation of the axis of maximum stiffness, measured with respect to the sagittal suture, was 36.276° ± 25.987° for the inner cortical table and 39.077° ± 21.6° for the outer table (Fig 5). Differences in angular orientation were not statistically significant (F = 0.077, p = 0.784).

**Fig 6:**
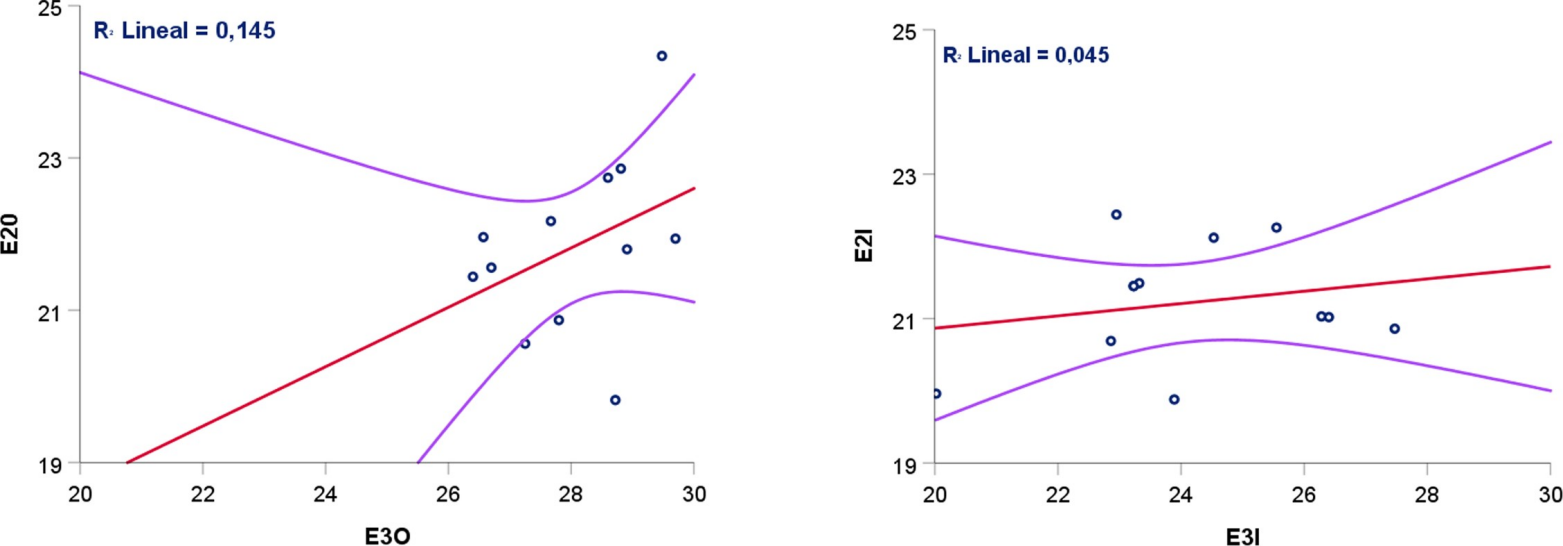
Scatterplot of the correlation between E_2_ and E_3_ for (A) outer and (B) inner parietal cortical plates.

Anisotropy, expressed as the ratio E_2_/E_3_, was not statistically different between inner (0.5938 ± 0.083) and outer (0.5814 ± 0.040) cortical plates (t = -0.497, p = 0.629). Moreover, Pearson´s correlation of E_2_ and E_3_ for outer and inner tables shows a low correlation for both cases (Fig 6).

## Discussion

### Elastic moduli

Overall, there were no significant differences in anisotropy (E_2_/E_3_) between inner and outer parietal cortical plates, suggesting the baboon cranial vault is orthotropic. Complementarily, there were significant differences among the three principal elastic moduli between the inner and the outer cortical tables (E_3_ > E_2_ > E_1_) of the parietal bone in baboons suggesting that both cortical tables are orthotropic, and indicating that their osteons are oriented in a relative uniform pattern [9]. Moreover, the principal elastic moduli were larger in the outer cortical plate than in the inner cortical plate (Table 1). These results are similar to previous results reported from humans [3]. Our results are also in agreement with previous works that reported an orthotropic condition of the outer cortical plate of the skull in *Macaca mulatta* [11], chimpanzees [8], and baboons [12]. In these previous reports, the reported elastic modulus values were lower that we reported here, because the previous studies averaged results of elastic moduli among parietal, frontal, and sphenoid bones. Complementarily, some authors have previously reported that the human cortical plates are transversely isotropic [4, 9, 24]. We believe that their results were affected due to the dynamic effect of the strain rate during the tension tests [24], the high average age (68.4 years) of the human subjects [4], and the lack of a pattern orientation when removing the specimens from the skull [9].

### Shear moduli

Our tests of a single baboon skull found that the three shear moduli were statistically larger in the outer parietal cortical table than in the inner table (G_31_ > G_23_ > G_12_), which replicates previous reports of human parietal cortical plates [3]. However, a previous study in chimpanzee external cortical plate of the skull reported a different order for the shear moduli: G_23_ > G_31_ > G_12_ [8]. Complementarily, a study in the outer cranial cortical plate in baboons kept on the different pattern for the shear moduli: G_23_ > G_31_ > G_12_ [12].

### Density

Our study showed that the outer table was more dense than the inner table, which replies earlier findings [3]. Although at least one study has suggested that bone density measurements alone have a limited influence on bone strength [30], we propose that including complementary measurements of bone quality, such as elastic moduli and shear moduli, provide a more through calculation of the overall ability of the outer table to resist external orofacial loads.

### Thickness

Our measurements of the thickness of the layers of the baboon´s parietal bone found that the outer cortical plate was significantly thicker than the inner cortical plate. These results are in accordance with previous studies that reported the same characteristic in 10 human parietal bones [3]. They reported 1.8±0.3 mm for the periosteal cortical (outer) plate and 1.7±0.3 mm for the endosteal cortical (inner) plate [3]. In the same direction, one study in 4 human embalmed calvaria reported 1.41±.2 mm for the inner table, 3.43±0.92 mm for the diploe, and 1.69±0.18 mm for the outer table [25].

**Fig 7:**
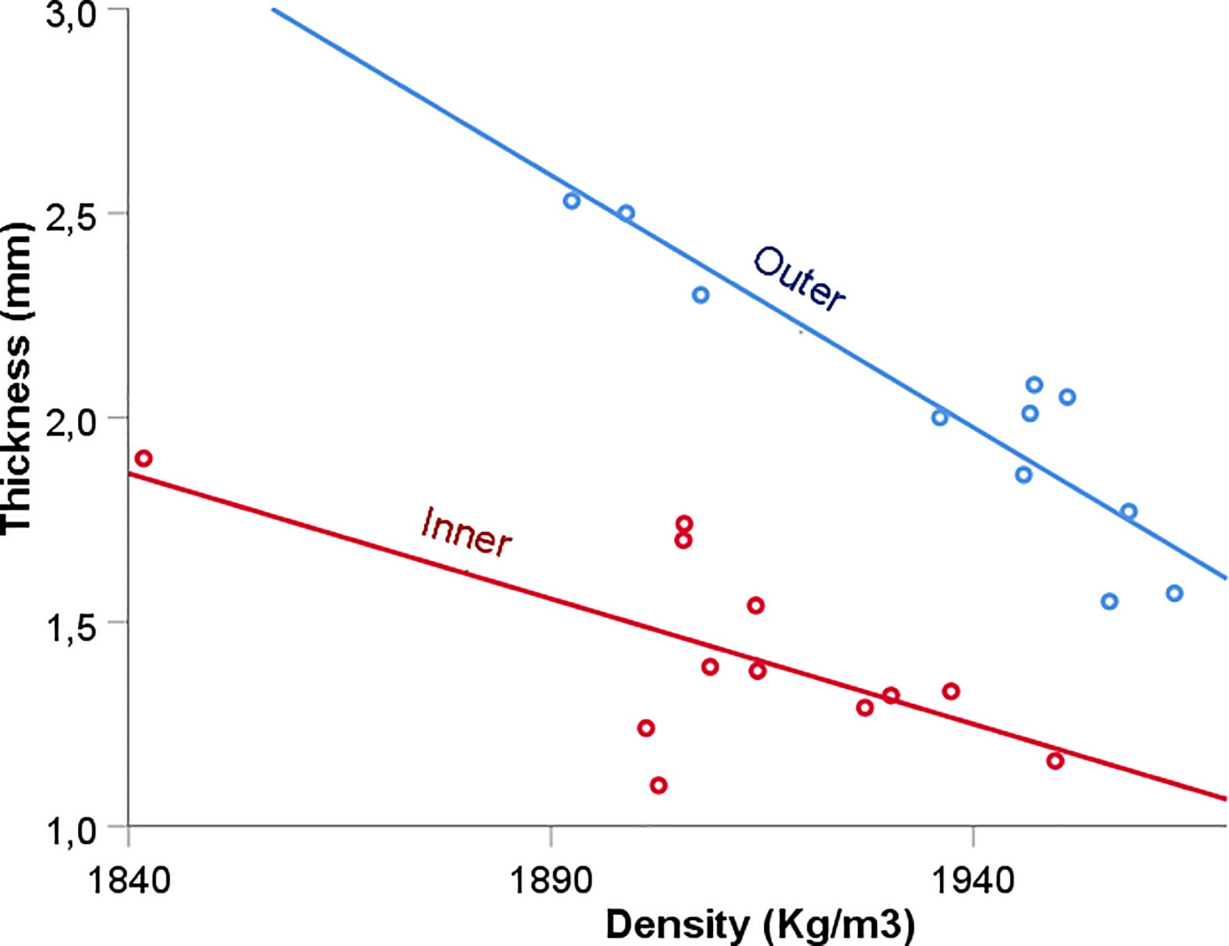
Scatterplot of the negative relationship between density and thickness for inner and outer cortical plates.

Thickness and density, which are two important factors in the overall strength of cortical bone, are negatively correlated with both the inner (R = -0.67) and outer (R = -0.93) cortical plates (Fig 7). Our results were in concordance with the outer cortical plate in *Macaca mulatta* skulls [11].

### Principal direction of stiffness

Principal directions of maximum stiffness were consistent in both inner and outer parietal cortical plates. The principal direction of maximum stiffness of the cortical plates is approximately 38° with respect to the sagittal suture in this individual baboon, suggesting a preferential anatomical strength in the antero-posterior orientation rather than in the medio-lateral direction of the cranial vault. This principal direction is correlated with diagonal linear fracture patterns reported for the parietal bone under compression forces applied to the human calvaria [17]. Opposite to our results, one previous study find no significant differences in the principal direction of stiffness between inner and outer cortical tables in humans [3].

### Mechanical properties

The three-layered cranial system is a brittle material, so a compression test is the most correct way to define its biomechanical characteristics because it is stronger in compression than in tension [21]. However, ultrasonic tests present a nondestructive option that provides the elastic characteristics of an anisotropic material in a dependable way. Although studies of the mechanical properties of the human cranial vault have generally reported transverse isotropy characteristics, with respect to the axis perpendicular to the calvarial surface [3, 4, 9, 15, 22, 25], other studies of the mechanical properties of the cortical skull in the Rhesus monkey (*Macaca mulatta*) [11, 23], baboons [12], and chimpanzees [8] have found orthotropic conditions of the cortical material. This difference among primate species could be due to the fact that the patterns of osteons in the cranial cortical bone may lead to a specific anisotropic condition in the cortical plates of the non-human primate skulls [9].

Mechanical properties of the three layered calvarium system in humans, baboons, chimpanzees, and macaques showed that extant primates skulls have different patterns of mechanical properties [12]. Thus, special attention is needed when trying to predict the biomechanical response of the craniofacial skeleton of extinct species [12]. The individual mechanical properties of the calvarial three-layered system are relevant to understand not only the functional response of the skull, but also its biomechanics, so clinical, functional, and anthropological models of the skull may be evaluated properly [31–33]. Additionally, understanding of the mechanical properties of the components of the cranial vault would make possible the development of better synthetic bone substitutes for the cranium [34].

### Functional implications

The dome shape of the skull is part of a complex group of functional cranial components [1], that have different mechanical properties linked to the environmental stress and the orofacial functions [3]. Complementarily, the three-layer system can be considered as an engineering sandwich structure that provides tensile and compressive properties via the internal and external cortical plates, with a central core that provides compressive and shear properties [24]. Functional masticatory and nuchal musculature activity produces tensile forces to the outer table and compressive forces to the inner table [35]. Thus, the outer plate is thicker, denser, and stiffer that the outer plate.

In the calvarial three-layered system, both the inner and outer tables provide the predominant stiffness to respond to the primary functional and accidental loads in the skull [36], whereas the trabecular bone of the central diploe is an energy absorbing lightweight structure that provides cushioning, shear strength, and separation between the cortical plates in order to increase the inertial characteristics (bending strength) that allow the three-layered structure to endure mainly bending loads [3, 36]. Although each of the three components of the engineering sandwich structure have different geometrical characteristics and mechanical properties, the combined material has the ability to response naturally to functional loads. However, it is important to consider that the mechanical properties of the whole three-layered system are different than the independent mechanical properties of each one of the three components.

### Numerical model implications

Averaged mechanical properties obtained from testing the whole calvarial system frequently assume that the cranial vault has a homogenous microstructure [7], proposing that the whole calvarial bone is an isotropic material. On the other hand, several finite element models (FEM) of the skull have been built using the information available for the engineering sandwich structure and the available mechanical properties. Consequently, most of the FEM of the skull can be grouped into one of four categories: (1) FEM of the skull built using a single isotropic material for an averaged layer in the entire calvarial structure [32]. (2) FEM of the skull including an anisotropic condition of the material for a single layered structure [37]. (3) Three-layered FEM structure with isotropic conditions for the material [31, 38, 39]. (4) Three-layered FEM system with anisotropic conditions for the vault [40]. However, how these different biomechanical assumptions of the three-layered system would affect mechanical responses in FEM simulations is not clear. New knowledge on the mechanical properties of the three-layered system of the skull will allow both enhance models of functional activities in extant primates, and the improvement of functional modeling of fossil hominids.

In conclusion: bone strength is the combination of both the morphology and the mechanical properties of a particular bone, suggesting its ability to endure functional loads as a sign of adequate adaptation. However, previous studies have found only low values of strains within the cranial vault from chewing and biting [41] suggesting that, in adult subjects, the main goal of the three-layered system of the cranial vault is provide a surface for the attachment of the nuchal and craniofacial muscles, and protect the brain from external traumatic loads. In particular, the arrangement and the differences in mechanical properties of the two cortical plates support their different functions. Lastly, the cortical layers of the skull in most primates is orthotropic, except in humans which has transversally isotropic characteristics. These differences in mechanical properties could be due to natural biological variances.

### Limitations

Although this study is limited to the mechanical properties of the two cortical plates of a single baboon´s parietal cortical bone, the results may be applicable to other three-layer cranial bones as the frontal, the temporal, the sphenoid, and the occipital. In addition, it will be important to study the mechanical contribution of the trabeculae diploe to the overall mechanical property of the primate parietal cortical bone. Although only one subject was evaluated, we believe our results provide adequate pilot data from which to generate future hypotheses about mechanical properties of cranial cortical bone in primates. Although several specimens from cross the parietal bone were evaluated, other bones of the cranium were not evaluated. This study did not considered differences in mechanical properties among subjects, the incidence of the gender and the age were not considered.

## Supporting information

S1 File Paired-data-2018 Excel file including all the measurements and processes to obtain the mechanical properties of the cortical plates. Time delay graphs from the oscilloscope are attached to the corresponding cells.
(XLSX)
